# Alginate-Based Edible Films and Coatings for Food Packaging Applications

**DOI:** 10.3390/foods7100170

**Published:** 2018-10-17

**Authors:** Tugce Senturk Parreidt, Kajetan Müller, Markus Schmid

**Affiliations:** 1 Chair of Food Packaging Technology, TUM School of Life Sciences Weihenstephan, Technical University of Munich, Weihenstephaner Steig 22, 85354 Freising, Germany; 2 Fraunhofer Institute for Process Engineering and Packaging IVV, Giggenhauser Straße 35, 85354 Freising, Germany; kajetan.mueller@hs-kempten.de; 3 Faculty of Mechanical Engineering, University of Applied Science Kempten, Bahnhofstraße 61, 87435 Kempten, Germany; 4 Faculty of Life Sciences, Albstadt-Sigmaringen University, Anton-Günther-Str. 51, 72488 Sigmaringen, Germany; schmid@hs-albsig.de

**Keywords:** edible coating, edible film, food packaging, alginate, crosslinking, additives, application method, food, transport mechanism

## Abstract

Alginate is a naturally occurring polysaccharide used in the bio industry. It is mainly derived from brown algae species. Alginate-based edible coatings and films attract interest for improving/maintaining quality and extending the shelf-life of fruit, vegetable, meat, poultry, seafood, and cheese by reducing dehydration (as sacrificial moisture agent), controlling respiration, enhancing product appearance, improving mechanical properties, etc. This paper reviews the most recent essential information about alginate-based edible coatings. The categorization of alginate-based coatings/film in food packaging concept is formed gradually with the explanation of the most important titles. Emphasis will be placed on active ingredients incorporated into alginate-based formulations, edible coating/film application methods, research and development studies of coated food products and mass transfer and barrier characteristics of the alginate-based coatings/films. Future trends are also reviewed to identify research gaps and recommend new research areas. The summarized information presented in this article will enable researchers to thoroughly understand the fundamentals of the coating process and to develop alginate-based edible films and coatings more readily.

## 1. Introduction

The principal roles of food packaging are to protect food products from physical, chemical, and biological influences by delaying food deterioration, retaining and prolonging the beneficial effects of processing, and maintaining the quality and safety of the foods with extending shelf life [1]. Broad external influences such as the development of international food markets, legal and technological requirements, raw material availability, consumer demands, etc. caused the food packaging area to change perpetually [2]. A total of 1.3 billion tonnes of municipal solid waste per year was generated in 2012, but it is expected to increase to 2.2 billion tonnes per year by 2025 [3]. Non-renewable, non-biodegradable packaging materials have serious environmental drawbacks. They have been considered a major source to the solid waste and environmental pollution by consumers and environment activists [4,5]. In order to solve this problem, companies and researchers have been working on ways to develop new packaging strategies with environmentally friendly, abundant biodegradable packaging materials made from renewable natural polymers [4,6]. Furthermore, the rapidly growing interest in the use of edible packaging can also be associated with a growing interest from consumers for minimally processed fresh-like foods with an extended shelf life and trend in improving the quality of food with edible barriers [7].

Edible films and coatings are thin layers of material (their thickness is generally less than 0.3 mm) used for enrobing the food product to replace or fortify the natural layers and can be consumed as a part of the product or with further removal [8,9]. Therefore, the materials used in the formulation should conform to the general food laws and regulations [10]. Additionally, the coatings and films should not affect the organoleptic properties of the food product negatively [6]. 

Edible packaging can be a superficial coating on the food or continuous layers between compartments/ingredients of the heterogeneous products (e.g., pizza, bakery fillings, and toppings) [8]. The coating can also be applied on individual pieces of the whole product, which have not been individually packaged due to practical arguments, such as fresh-cut melons, kiwis, strawberries, nuts, beans, pears [11]. 

Edible films and coatings can be used to overcome many obstacles involved in the marketing of foods [12]. These functions can be specified as retarding moisture, gas, solute and oil migration, improving structural integrity, retaining volatile flavor compounds, conveying food additives [12]. In addition, they improved the aesthetic appearance by minimizing the development of physical damage, hiding scars, and improving surface shine [13,14]. For instance, hot-melt paraffin waxes have been used to coat citrus fruits to retard moisture, edible collagen casings have been used for sausages to provide structural integrity and apples have been coated with wax to improve surface shine and prevent physical damage.

The required features expected from edible films and coatings can be assigned by the specific characteristics of the product and changes during production, transportation, and storage periods. Despite providing a barrier, the non-edible packaging is still essential for edible coated food products due to hygienic reasons [8]. Nevertheless, combining edible films and coatings with traditional packaging would likely reduce the non-biodegradable packaging waste of processed foods and environmental effluence [12,15,16].

Although edible coating and edible film terms have been used interchangeably or as synonyms in some sources; their application on the food products constitutes their main difference [9]. Edible films are stand-alone wrapping materials which can be cut and placed on the food product separately due to having enough integrity; on the other hand, edible coatings form a thin layer on the product directly subsequent to the application [8,9,17,18]. Therefore, although produced from the same gelling agent, the characteristics of edible films and coatings can be very different [9]. 

Alginate-based food coatings and films attracted widespread interest. A wide range of scientific research has been published in the literature. This overview summarizes the literature information with dividing into categories in a layout:

1. General information about alginate and gel formation
2. Lists of additives incorporated into the alginate-based edible films and coatings in the literature
3. Types of film production and coating application
4. Sums up the research findings on alginate coated fruits-vegetables, meats, poultry, seafood, cheese
5. Transport of the products’ molecular components
6. Future trends

Our present study can be used as a guide for researchers both in the academy and industry who plan to work on alginate-based coatings, will select the components of their formulations, and plan their future studies and experiments. 

## 2. Film-Forming Materials 

**Figure 1:**
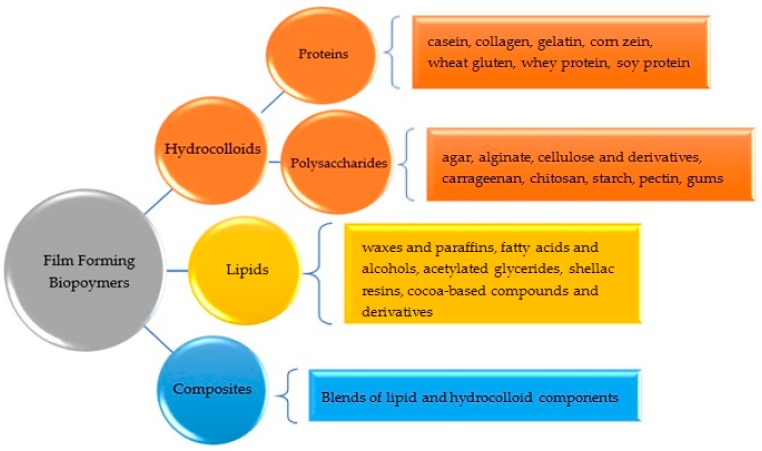
The film-forming biomaterials that have been studied extensively for the formation of edible coatings and films (Donhowe and Fennema [12], Embuscado and Huber [25]).

Film-forming biopolymers are generally classified according to the type of film-forming material, which form cohesive and continuous matrices [8]. These are hydrocolloids (polysaccharides and proteins), lipids and composites (Figure 1) [12,19]. Hydrocolloids are composed of hydrophilic polymers of microbial, vegetable, animal, or synthetic origin [15]. Mostly, they are large molecules with many hydroxyl groups [15,20]. Hydrocolloid film applications do not target to control water vapor migration due to their hydrophilic nature [12]. However, the continuous polysaccharide film can be referred to as a sacrificial moisture agent. That is, moisture evaporates from the film instead of the food surface [21,22,23,24]. Subsequent to the desiccation of the coating film, the food product would lose its moisture [24].

In general, polysaccharides are used as gas barriers; lipids reduce water transmission, while proteins provide mechanical stability [9]. The main disadvantages of lipid-based films and coatings are their opaqueness, fragility, and instability (rancidity), on the other hand, hydrocolloid films and coatings have a more neutral taste [6]. Composites are formulated using both lipid and hydrocolloid components, which are incorporated into the formulation in order to benefit from their advantages together [12]. Composites can be formed as a bilayer or as a conglomerate [12]. 

## 3. Alginate

Alginates are naturally occurring, indigestible polysaccharides commonly produced by and refined from various genera of brown algae (mainly *Laminaria hyperborean*, *Macrocystis pyrifera*, *Ascophyllum nodosum*; lesser extent *Laminaria digitate*, *Laminaria japonica*, *Eclonia maxima*, *Lesonia negrescens*, *Sargassum* sp.) [26,27,28,29,30]. Some bacteria such as *Azotobacter vinelandii* or mucoid strains of *Pseudomonas aeruginosa* also synthesize alginate like polymers as exopolysaccharide (i.e., extracellular polymeric substances, EPSs) [29,31]. Alginate production from Marine algae [32] and *A. vinelandii* [33] are explained elsewhere.

**Figure 2:**
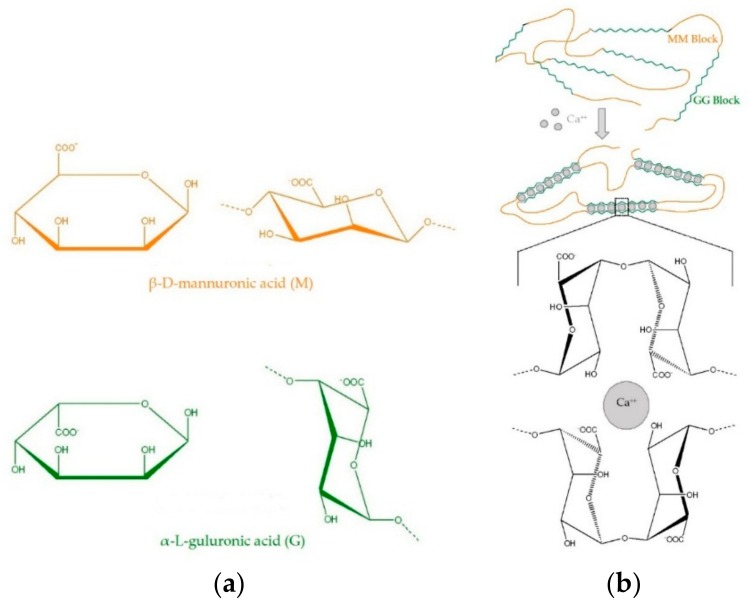
The structural formulae of monomeric units in alginate and the schematic representation of the egg-box model (**a**) Left hand side: Haworth conformation; right hand side: Chair conformation (**b**) Gelation of poly L-guluronate blocks (G Blocks, 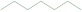) with Ca^2+^ (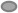) (Peteiro [32], Lee and Rogers [38]).

The molecular structure of alginates is composed of unbranched, linear binary copolymers of β-d-mannuronic acid (M) and α-l-guluronic acid (G) residues linked by 1–4 glycosidic bonds (Figure 2a) [27,28,34,35]. An algal alginate structure could be separated into three fractions (three uronic acid blocks): These are homopolymeric regions of M and G blocks, and alternating MG blocks containing both polyuronic acids [27,35,36]. Bacterial alginates have O-acetyl groups, while they are not present in the structure of algal alginates [37]. Additionally, bacterial alginates have higher molecular weights compared to the algal polymers [33].

The source of the alginate affects the ratio of M and G residues, which have an impact on the physical and chemical properties of the alginate, as well as the viscosity of the coating solution and thickness on the product [28,36,39]. Martinsen, et al. [40] characterized the relationship of physical properties of alginate gel beads with a polymer composition, sequential structure, and molecular size of the several different alginate sources. 

Alginic acid was first discovered and isolated by Dr. E.C.C. Stanford in 1881 [35]. Alginates (i.e., sodium alginate (E401), potassium alginate (E402), ammonium alginate (E403), and calcium alginate (E404)) are monovalent salts of alginic acid (E400) [27,41,42]. Alginic acid and calcium alginate are insoluble in water while sodium alginate, potassium alginate, and ammonium alginate are water-soluble polymers [42,43]. They have a limited solubility at low pH values [44]. The solubility of different types of alginates in numerous solvents and solutions were listed by Kimica Corporation [45].

The U.S. Food and Drug Administration (FDA) classifies food grade sodium alginate as GRAS (generally regarded as safe) substance in Title 21 of the Code for Federal Regulations (CFR) and lists its usage as an emulsifier, stabilizer, thickener, and gelling agent [46]. The European Commission (EC) listed alginic acid and its salts (E400–E404) as an authorized food additive [41]. 

Alginate is widely used in various industries such as food, beverage, textile, printing, and pharmaceutical as a thickening agent, stabilizer, emulsifier, chelating agent, encapsulation, swelling, a suspending agent, or used to form gels, films, and membrane [28,47]. Sodium alginate is the most common salt of alginate [48]. 

## 4. Crosslinking

It is widely well-known that alginate is polyuronide, a natural ion exchanger [49]. The charged state of alginate is beneficial for film formation. In the absence of bivalent ions, alginate can be only used to increase viscosity [38]. However, the addition of certain bivalent cation into the alginate solution leads to a gel formation through ion exchange [42,47]. The affinity of alginate for the alkaline earth metals increases in the order Ca^2+^ < Sr^2+^ < Ba^2+^ [38,49]. Monovalent cations and Mg^2+^ ions do not form a gel [50]. Even though divalent cations such as Pb^2+,^ Cu^2+^, Cd^2+^, Co^2+^, Ni^2+^, Zn^2+^, and Mn^2+^ can also induce gelation, their toxicity limits their utilization [51]. 

Alginate gel formation is rather a complex process. The proportion and length of the guluronic acid block (G-blocks) in the polymeric chain, the capacity to bind the number of divalent ions, the type of gelling ions and gelling conditions affect strongly the hydrogel properties of alginate [39,52,53,54]. The introduction of divalent cations (usually Ca^2+^ ions) to the system induces conformational changes in alginate such as alignment of the G-blocks and the formation of the egg-box model [54] due to bounding of calcium ions between two chains and forming divalent salt bridges (Figure 2b) [39,40]. A higher amount of G-Blocks will create rigid and dense gels, while a higher amount of M-Blocks will build flexible, porous gels [54,55,56,57]. Therefore, the diffusional resistance of gels containing predominantly high polyguluronic alginate content against high-molecular-weight-compounds is high [56,57]. Besides, the study by Olivas and Barbosa-Cánovas [58] revealed that films containing higher proportions of G-Block (in other words, lower M/G ratio) showed better moisture barrier characteristics. 

There are two types of procedures for the incorporation of gelling ions into the alginate solution to form hydrogel (i.e., external and internal gelling modes) [59,60]. (1) For external gelation (the traditional method), alginate solution is directly exposed to the solution of gelling ions, Ca^2+^ instantaneously reacts with carboxylic groups of guluronic acid residues and hydrogel is formed irreversibly due to the diffusion of ions [52,59,61]. (2) On the other hand, the internal gelling method consists in incorporating an insoluble source of gelling ions with alginate solutions and afterward, the gelling ions are released by processes that lower the pH: the addition of organic acids or slowly hydrolyzing lactones [52,59]. When these two methods are compared, internal gelation forms more homogeneous but less dense gel matrices with larger pore sizes compared to the external gelation due to the displacement of Ca^2+^ by H^+^ with the acid addition [60,61]. 

According to Mancini and McHugh [53], gel formation by cooling of the hot solution, which contains all the components, can be evaluated as the third method to initiate the controlled alginate gelation. Due to the thermal energy that the alginate solution possesses, a calcium-induced hydrogel formation can only take place after cooling [53].

The source of calcium ions (i.e., calcium chloride, calcium lactate, calcium gluconate, calcium nitrate, and calcium propionate) has an influence on gel formation. Allen et al. (as cited in Reference [62,63]) reported that crosslinking with calcium chloride generated stronger alginate gels compared to calcium gluconate, calcium nitrate, and calcium propionate. The calcium source also affects the kinetics of gelation; in other words, the rate of gelation and Ca^2+^ concentration is positively correlated [38]. Calcium chloride has the highest solubility (75 g/100 mL) followed by calcium lactate (8 g/100 mL) and calcium gluconate (3 g/100 mL) at 20 °C [38]. The steady-state gel strength was reached fastest by calcium chloride followed by calcium lactate and calcium gluconate [52]. On the contrary, according to the study by Chrastil [64], the gelation kinetic constants did not depend on the calcium source. However, the strength of the formed gel and the resistance against calcium diffusion are not dependent on the calcium source type [38,52]. 

Despite its high solubility, calcium chloride is not an attractive calcium source due to imparting a bitter taste on the food [38]. On the other hand, calcium gluconate and calcium lactate can be used in coating applications where the taste attributes are important [38].

In situ gelation of alginate, and strongly homogeneous structure forming are also an interest of biotechnology (e.g., tissue engineering, immobilization of cell and enzyme systems, carriers for drug delivery system). A technique to prepare homogeneous alginate gels with a slow release of calcium ions has been reported [65,66]. In a similar approach, Kuo and Ma [67] used calcium carbonate D-glucono-δ-lactone (CaCO_3_ -GDL) or calcium sulfate dihydrate (CaSO_4_.2H_2_O -CaCO_3_ -GDL) as a gelation agent due to their very low solubilities, which allows for a uniform distribution of the CaCO_3_ in the alginate solution and, therefore, a more controlled and more uniform gel formation. In their subsequent research, the dimensional stability (swelling tendency of the gel due to an increased tendency of the carboxyl and hydroxyl groups to interact with water molecules and osmotic pressure) was controlled by controlling the calcium ion concentration of the external aqueous environment, crosslinking density, and polymer concentration of the gel and chemical composition of alginate [68]. 

It was pointed out by Pavlath, et al. [69] that two type of reactions take place when an alginate film/coating is immersed in crosslinking calcium solution: diffusion of the multivalent ion which induces formation of calcium linkage with the carboxyl groups for the insolubilization of the alginate film, and the dissolution of alginate by the solution [58]. The increased concentration of the multivalent ion diminishes the dominance of the dissolution process [69]. Rhim [70] also noticed the same phenomena and added that the method of CaCl_2_ treatment affected the thickness of the film, i.e., direct addition of the crosslinking agent lead to thicker films compared to immersing the alginate films into a crosslinking solution. 

The properties of the formed film are also dependent on the mixing temperatures; although mixing at ~20 °C caused an immediate gel formation that could not be cast, mixing at ~50 °C formed a viscous solution which could be poured into the frames [69]. 

The degree of crosslinking affects the swelling ability of the 3D structure of the alginate in the solvent, and we end up with a decrease in the permeability to various solutes and being used in drug controlled release systems [71]. In the study by Zactiti and Kieckbusch [71], the degree of swelling decreased (in other words, the crosslinking was increased) with the increasing concentration of Ca^+2^ ions in the crosslinking solution and, therefore, the solubility and elongation of the alginate films decreased, while the tensile strength increased. Similarly, Rhim [70] observed that an increase in the CaCl_2_ concentration caused an increase in the tensile strength and a decrease in the percentage elongation at break. 

The literature on the effects of calcium chloride dipping alone (without coating) has shown that CaCl_2_ can be used as an effective firming agent due to the ability of calcium to bind cell wall polymers, maintain its structure, and diminish the water solubility of pectic substances with forming calcium pectate [72,73,74]. Improvement in firmness increases with increasing concentration of CaCl_2_, however, this is independent from the dipping time [75]. 

## 5. Additives

The mechanical, functional, organoleptic, and nutritional characteristics of edible films and coatings can be modified with the incorporation of various natural or chemical additives [12].

### 5.1. Plasticizers

The mechanical properties of biodegradable packages can be improved by the plasticization of the polymer-network with plasticizers, which are generally non-volatile and miscible with the polymer. The primary objectives of the plasticizers are increasing the free volume or molecular mobility of polymers, decreasing the intermolecular forces, bestowing flexibility, reducing brittleness, improving tear impact resistance, and regulating the flow of the coating material [26,76,77,78,79]. Furthermore, the plasticizers should have the same solubility properties with the polymer in the solvent system (to prevent plasticizer or polymer separation during film/coating application), possess a high boiling point, and should be able to change the physical and mechanical properties of the substance when incorporated into the formulation [6,76]. 

Water is the most common and most effective plasticizer; still, the plasticizing effect of water in hydrophilic biopolymers is difficult due to the dependency of the environmental conditions such as relative humidity and temperature [6,8]. Apart from water, glycerol, sorbitol, acetylated monoglyceride, polyethylene glycol, sucrose, etc. have been used as plasticizers in food coating studies [12]. The addition of hydrophilic plasticizers to the formulation generally promotes water vapor permeability (WVP) and influences the mechanical properties of the coating material [12,80]. Therefore, the type and the quantity of the plasticizer are very important in the designing of the edible coating formulation. Parris, et al. [81] stated that based on the total solid content of the film, the plasticizer amount should be determined between 10% and 25% as lower concentrations cause brittleness whereas higher concentrations lead to stickiness. 

Glycerine and sodium lactate lead to stronger and more elastic alginate-based films compared to sorbitol, which was stiffer [81]. However, sorbitol added films exhibited better water vapor barrier properties at the same concentration due to being less effective in reducing intermolecular hydrogen bonding between polymer molecules [81]. Jost, Kobsik, Schmid, and Noller [80] compared the effects of glycerol and sorbitol addition to alginate films in terms of their mechanical properties and determined that both plasticizers decreased equilibrium moisture content and porosity. On the other hand, the incorporation of glycerol caused higher WVP and oxygen permeability, while sorbitol did not alter barrier properties [80]. Similarly, Olivas and Barbosa-Cánovas [58] analyzed the effects of different plasticizers (glycerol, sorbitol, polyethylene glycol (PEG), and fructose) on WVP and the mechanical properties of calcium alginate films in two different RH values. WVP increased in the order of fructose, sorbitol, glycerol, and PEG incorporated films. Plasticizers induced a sharp increase in moisture content in the moisture sorption isotherm graphs and modified the mechanical properties with an increasing tensile strength.

In several studies, glycerol has been used as a plasticizer, particularly for alginate films and coatings. However, the amount used is variable. Rojas-Graü, et al. [82] reported that the water vapor resistance (WVR) of alginate coatings increases with an increasing glycerol concentration up to 1.75% (*v*/*v*) in the formulation; however, WVR decreases at higher concentrations of glycerol. Likewise, Azarakhsh, et al. [83] observed the same type of effect during the optimization of the alginate coating formulation and determined the amount as 1.16% (*w*/*v*). On the other hand, Tapia, Rojas-Graü, Carmona, Rodríguez, Soliva-Fortuny, and Martin-Belloso [39] determined that glycerol concentrations above 1.5% (*w*/*v*) decreased the WVR.

High wettability and uniform spreading ability of the edible coating on the targeted food product are desired characteristics of edible coatings while designing the formulations [84,85,86]. Therefore, the effects of the components on the surface tension of the coating solutions are an important factor. Yet, glycerol and sorbitol do not have a significant effect on the surface tension of the solutions due to not being tensio-active substances [87,88].

High amounts of plasticizer (>10%) have also been incorporated in alginate-based edible coating formulations in the literature [89,90,91,92,93].

Some studies combined more than one plasticizer in order to overcome the brittleness of the alginate films and coatings. Fan, et al. [94] chose glycerol, palmitic acid, β-cyclodextrin, and glycerol monostearate in film formulation. Su Cha, et al. [95] used a 1:1 concentration of polyethylene glycol and glycerol combination.

### 5.2. Surfactants

Adhesion on hydrophobic, rough surfaces and obtaining a uniform edible coating can be very difficult due to the low surface free energy of the surface [9]. Addition of surface active agents (surfactants) are the key ingredients to increase the wettability of the product and improve the adhesion of the coating material [88]. Moreover, with a reducing superficial water activity, surfactants and emulsifiers had a decreased rate of moisture loss when they were incorporated into the coating formulation [96].

The major characteristics of the surfactants are being present at the surface of the interfaces (liquid-air, liquid-liquid, liquid-solid) at higher concentrations compared to the bulk of the liquid [97]. Surfactants can be classified based on two characteristics: charge type of the surface active part and the chemical structure of the hydrophilic groups [97]. Accordingly, these groups are; anionic (negatively charged), non-ionic (no charged group), cationic (positively charged), and amphoteric (can be positively or negatively charged, or both, depending on the circumstances) surfactants [97].

The uniform spreading ability of the coating on the targeted product is a very important effectiveness indicator of an edible coating. Therefore, researchers checked the surface free energy of the food products, the surface tension of the edible coatings, and spreading coefficient (W_s_) while designing their coating formulations. Senturk Parreidt, Schott, Schmid, and Müller [88] characterized the alginate-based coating formulations with various concentrations and types of surfactants (0–5% tween 40, tween 80, span 80, span 60, and soy lecithin).

### 5.3. Antimicrobials

The addition of antimicrobial and antioxidant agents to the edible coatings and films is beneficial compared to their direct application to food products due to providing the opportunity of gradually releasing the agents and maintaining a critical concentration for a prolonged period [26,71,98,99]. In contrast with the migration of antimicrobials from the coating, the direct addition of antimicrobials to food will cause immediate microbial inhibition while the recovery of the injured cells and the later growth of the undestroyed cells may cause quality losses and/or foodborne diseases [100].

A great variety of antimicrobial agents have been incorporated into alginate-based edible films and coatings (Table 1). The summarized results show that alginate forms an effective base for antimicrobials to decrease the microbial load of coated food products.

Antimicrobial properties, mode of action, and the potential uses of essential oils (EOs) have been revealed in various studies in the literature [101,102,103,104]. These natural preservatives have also been added into edible films and coatings to introduce antimicrobial properties [105]. The limiting factor of their usage is their strong flavor, which originates from the phenolic compounds (i.e., abietane diterpenes, carnosol, ursolic acid) they contain [105].

### 5.4. Antioxidants

Antioxidants have been defined by FDA as “substances used to preserve food by retarding deterioration, rancidity, or discoloration due to oxidation” [144]. Autoxidation comprises of a free radical chain mechanism in which unsaturated fatty acids react with free radicals [145]. Apart from autoxidation, lipid quality deterioration can arise from photooxidative conditions, oxidation via lipoxygenase-assisted process or oxidation under high temperatures [145].

Coatings can also serve as carriers of antioxidative substances to protect against discoloration, degradation and oxidative rancidity [96]. Table 2 lists antioxidant agents used in alginate-based edible films and coatings. The application of an edible coating with incorporated antioxidants decreases the oxidation successfully due to the gas barrier properties of alginate coating and the synergistic effect between two factors.

Phenolic antioxidants, which have often been incorporated into alginate-based coatings and films, do not work as oxygen absorbers but prevent the formation of fatty acid-free radicals and, therefore, their absorbance of oxygen in autooxidation [128].

### 5.5. Antibrowning Agents

Color is a critical quality parameter. Browning reactions during the shelf life of fresh-cut fruits and vegetables result from both enzymatic and non-enzymatic oxidation of phenolic compounds [155,156,157]. Fresh-cut fruits and vegetables undergo minimal processing operations such as cutting, peeling, trimming, coring etc., that disrupt cellular compartmentalization, thus, browning proceeds much rapidly [156]. It is well known that browning in fruits and some vegetables mainly originate by the enzymatic oxidation (polyphenol oxidases) of phenolic compounds [156,157]. Polyphenol oxidase requires oxygen to start browning reactions; therefore, implementing an oxygen barrier can be beneficial in preventing browning [156,157]. Additionally, antibrowning agents are utilized against oxidative rancidity, degradation, and enzymatic browning in fruits and vegetables [82].

Edible coatings can be effective carriers for antibrowning agents withholding the agents on the surface of the cut tissues [79]. Antibrowning agents are commonly incorporated in crosslinking solutions and applied after the adhesion of the edible coating solution on the surface of the fresh produce [79]. Rojas-Graü et al. and Raybaudi-Massilia et al. incorporated N-acetylcysteine [79,82,109] and glutathione [82,109] into a calcium chloride bath to control the browning of fresh-cut apples and accomplished to keep apple wedges free from browning during storage. Likewise, Oms-Oliu, Soliva-Fortuny, and Martín-Belloso [147] used the same agents to avoid the browning of fresh-cut pears. Azarakhsh, Osman, Ghazali, Tan, and Mohd Adzahan [111], Montero-Calderón, et al. [158] and Sarengaowa, Hu, Jiang, Xiu, and Feng [107] added ascorbic and citric acid into a calcium chloride solution for coating fresh-cut pineapples and apples.

### 5.6. Flavors, Pigments, Nutritional Improvements

The organoleptic properties of the coated products can be improved with the addition of some ingredients such as flavorings, coloring agents, sweeteners, spices, and seasonings to the coating matrix [6,11,26]. Hambleton et al. encapsulated n-hexanal [159,160] and D-limonene [160] in alginate-based emulsified films with the help of lipid addition. Several nutritional additives such as vitamins, minerals, and probiotics have been incorporated in the edible films and coatings without damaging the integrity of the product [26]. Lipids (especially sunflower oil) have been extensively incorporated into alginate-based coating formulas and have increased their water resistance characteristics [82,83,147,158,161]. However, in addition to water resistance improvement, consumption of vegetable oils has also some health benefits. For instance, the European Food Safety Authority (EFSA) and EU Vegetable Oil & Protein Meal Industry (FEDIOL) declare that sunflower oil is rich in unsaturated, polyunsaturated fatty acids, and vitamin E content [162,163,164]. Although sunflower oil has been generally preferred as a lipid source in the literature, other vegetable oils have also been incorporated into the formulations. Ramana Rao, et al. [165] prepared a composite coating consisted of alginate and olive oil enriched with ascorbic and citric acid. Bazargani-Gilani [127] added resveratrol as a dietary supplement into a calcium-alginate gel. Tapia, Rojas-Graü, Rodríguez, Ramírez, Carmona, and Martin-Belloso [161] incorporated viable *Bifidobacterium lactis* Bb12 for a probiotic coating of apple and papaya cylinders, while Rößle, et al. [166] added prebiotics, oligofructose, and inulin to the coating of fresh-cut apples. Nair, Saxena, and Kaur [146] enriched the alginate-based coating with a pomegranate peel extract to enhance the phytochemical level of quava. Artiga-Artigas, Acevedo-Fani, and Martín-Belloso [138] added a mandarin fiber with prebiotic properties to the alginate coating and increased the nutritional value of the low-fat cut cheese.

## 6. Application Methods

Film formation methods and conditions of coating processes have important impacts on the physical properties of the formed film [26]. Uniform and defect-free (i.e., no air bubble and mechanical damage) film forming is very crucial to optimize its functionalities [26].

### 6.1. Film Formation

The mechanisms of edible film formation are listed below [10]:

1. Simple coacervation: The precipitation or phase change of the hydrocolloid, which is dispersed in water, is achieved following to (i) the solvent evaporation process (i.e., drying); (ii) incorporation of hydrosoluble non-electrolyte (in which the hydrocolloid is not soluble, e.g., ethanol); (iii) the pH adjustment with the addition of electrolyte, which impel salting out or cross-linking.
2. Complex coacervation: The precipitation of the polymer complex is achieved by mixing two hydrocolloid solutions, which have opposite electron charges.
3. Gelation or thermal coagulation: Precipitation or gelation is accomplished by heating of the macromolecule which causes its degradation (e.g., proteins such as ovalbumin) or the cooling of hydrocolloid dispersion (e.g., agar, gelatin).

The production principles of shelf-standing film techniques are similar to those for thermoplastic films: solvent casting and extrusion, although the conditions are different [26].

#### 6.1.1. Solvent Casting

It is the most frequently used film-forming technique, which consists of spreading of water or water-ethanol solutions/dispersions on a suitable surface and later air drying during several hours in a ventilated oven such as infrared drying chambers [26,167]. Following the evaporation of the solvent, the film is peeled from the surface without any damage. The structure of the film is depended on the composition of the casting solution, wet casting thickness, temperature and relative humidity of the drying conditions [26]. Rapid drying of the casting solution should be avoided due to reducing the solvent concentration very fast, and therefore limiting the mobility of the polymer chain and development of intermolecular interactions in the film [26].

#### 6.1.2. Extrusion

The extrusion technique is based on the thermoplastic properties of the polymers [167]. Subsequent to the plasticizer addition, the solution is heated above its glass transition temperature under low water content conditions [26,167]. The extrusion method is preferred in commercial applications due to the lack of solvent addition and evaporation steps [167].

The co-extrusion technique can be utilized to form multilayer films. However, due to the differences in the chemical-physical characteristics of each film-forming material, mechanical, optical, and barrier defects can come into existence [26].

### 6.2. Coating Application

Application method and the ability of the coatings to adhere to the food surface are the two important characteristics regarding edible coatings [167].

#### 6.2.1. Dipping

Food products are usually coated by a dipping or spraying technique, in which a thin film was formed on the surface that acts as a semipermeable membrane to control moisture loss and gas transfer [168,169].

This method consists of four steps: sample immersion into the alginate dispersions followed by withdrawing the sample and draining the excessive film-forming solution covering the product. Then, a second immersion of the alginate coated sample into the crosslinking bath to achieve the gel formation and draining of the excessive solution [170]. The dipping process is generally very short, therefore the evaporation from solvents in the coating and crosslinking solutions are neglected [171]. The duration of the dipping and draining times differ from each study, but it is generally between 30 s to 5 min. The main advantage of the method is its total coating even around complex and rough surfaces [172].

Due to the hydrophilic surface of the cut surfaces of the food products, a good adhesion cannot be easily accomplished by the simple dipping of the food product into the coating material. Multilayer coating or the layer-by-layer (LbL) technique has been used to minimize the sticking difficulty of the coating on the hydrophilic surface of the cut surfaces [26,110,173,174]. The process is based on the dipping of food product into different coating solutions that contain oppositely charged polyelectrolytes to achieve physical and chemical bonding to each other [110,174]. Currently, the LbL technique is applied mainly on fruits and vegetables [175].

The dipping method forms thick coatings. Additionally, Duan, Wu, Strik, and Zhao [92] pointed out that dipping might have diminished the efficacy of the coating application due to the dilution and dissolving effect.

#### 6.2.2. Spraying

The spraying method is another conventional technique used to form a semi-permeable membrane on the surface of food products [89]. The spraying system distributes the coating solution through the formation of droplets over the targeted food surface area with the help of nozzles [172]. The spraying technique needs less amount of coating material to achieve good coverage due to high spraying pressure (approx. 60–80 psi) [176]. The other advantages of this method are the uniform coating, control of thickness, possibility of multilayer applications, avoidance of coating solution contamination, temperature control of the solution and enabling of working with large surface areas [172].

The spraying solution should not have a high viscosity. Spray-flow characteristics are dependent on (i) liquid properties (i.e., density, viscosity, surface tension), (ii) operating conditions (flow rate, air pressure, etc.), and (iii) system conditions (nozzle design, spray angle, etc.) [172,177].

Earle and McKee [22] sprayed the dispersion containing water-soluble algin and, later, a gelling agent on the freshly slaughtered hanging animal carcasses. Researchers determined the viscosity of the solution as the critical point of the vertical spraying application to have a uniform adherence to the alginate film. Papajová, Bujdoš, Chorvát, Stach, and Lacík [59] designed a setup to prepare externally gelled planar alginate hydrogels with the airbrushing aerosols of a gelling solution. Amanatidou, Slump, Gorris, and Smid [148] applied a different spraying procedure. Carrot slices were initially dipped in CaCl_2_ solutions and, following the drying, an alginate-based solution was sprayed on the surface of the slices.

#### 6.2.3. Vacuum Impregnation

The Vacuum Impregnation (VI) method has been used for the enrichment of the product with vitamins and minerals in food research. Recently, studies showed that VI coating can form a thicker and more effective film with the incorporation of solutes into the air containing porous food matrices such as fruits and vegetables [178,179].

The VI method consists of the same dipping-draining steps, which were explained in the dipping subsection. However, instead of dipping tanks, the samples are submerged into two airtight vacuum chambers connected to vacuum pumps. Subsequent to the vacuum application, products are subjected to atmospheric restoration while they remain immersed in the coating solution under atmospheric pressure. Vacuum period, vacuum pressure and atmospheric restoration time are important parameters in the VI process [180].

## 7. Alginate-Based Coatings and Film Applications

There are many potential uses of sodium alginate-based edible films and coatings.

### 7.1. Fresh-Cut Fruits and Vegetables

Natural barriers (i.e., cuticle, skin, rind, etc.) that surround the whole and intact fruits and vegetables, protect the products against microbial contaminations and quality losses [181,182]. Lately, fresh-cut fruits and vegetables attract consumers’ attention due to increased awareness of healthy eating habits and having less time for food preparation [96,183]. The preferences of the consumers change in the direction of convenient consumption without any loss of quality characteristics [184]. Minimally processed fresh cut fruits and vegetables are subjected to preparation steps such as washing, peeling, cutting, slicing, coring, which remove these barriers, induce lesions of tissues, damage the integrity of the fruit, and cause wounding stress [79,106,185]. Thus, the product becomes vulnerable to contamination, enzymatic browning, undesirable volatile formation, and alterations in texture [183]. Deteriorative effects, as well as the growth of spoilage and pathogenic bacteria, can be prevented by enhancing the natural barriers or replacing it with artificial barriers surrounding the product such as an edible film and coating applications [182,186,187].

The delay of respiration and physiological processes are requisites for the shelf life extension of fruits and vegetables [10]. In this manner, coatings and films with the ability to modify the gas transport have the potential for applications in fresh produce coating [10,13,188]. In particular, polysaccharide-based coatings have been used to reduce the respiration of fruits and vegetables due to their selective permeabilities to the O_2_ and CO_2_ gases [27]. The swelling ratio and water solubility of alginate films are very important properties in case of fresh-cut fruits with high moisture surfaces. Tapia, Rojas-Graü, Rodríguez, Ramírez, Carmona, and Martin-Belloso [161] stated that alginate films have a resistance to being dissolved in water and, therefore, have the potential for coating high moisture fresh-cut fruits.

The studies conducted with alginate coated fresh/fresh-cut produce are shown in Table 3.

### 7.2. Meats, Poultry, and Seafood

There are various challenges associated with meats, poultry, and seafood products throughout their shelf life. They can be defined as moisture loss and its effects on texture, color, and flavor; unappealing dripping of the product juice (purge losses); lipid oxidation and brown discoloration; microbial spoilage; volatile flavor loss and/or gathering foreign odor [62,201,202].

In the patents, Earle et al. designed an alginate-based coating formulation commercially known as Flavor-Tex^®^ [203,204]. Meat, seafood, and poultry products were immersed into an aqueous dispersion of water-soluble algin and carbohydrate comprising mono and/or disaccharide sugar and gelatinized with a CaCl_2_-CMC solution (the addition of CMC to the calcium crosslinking bath reduced the gelling time and required concentration of the CaCl_2_) [203,204]. Lazarus, West, Oblinger, and Palmer [23]; West, Lazarus, Oblinger, and Palmer [24]; and Williams, Oblinger, and West [114] evaluated the effects of Flavor-Tex^®^ on lamb carcasses and beef pieces/steaks. Although calcium alginate coating acted as a sacrificing agent rather than a moisture barrier, maintaining a lower water activity (a_w_) on the surface together with the toxic effect of CaCl_2_ lead to lower microbial counts [23,114]. Nevertheless, the coating helped to stabilize the meat color, reduced shrinkage and obtained the same sensory scores compared to the uncoated meat samples [23,114]. Flavor-Text was also used to coat pork patties for solving the flavor and texture problems of precooked meat products [205]. Coating decreased the oxidative rancidity, cooking losses, and increased meat tenderness. To the contrary of the findings presented previously [23], calcium alginate coated row and precooked products received the highest sensory scores, which indicated that the higher structural integrity given by the coating was favored by consumers [205].

The usage of gelatinized alginate in the block freezing process of fishery products was patented in Norway, 1956 [206]. By this means, the detrimental effects of direct water contact on the products were eliminated [206].

Studies on alginate coated meat, poultry, and seafood products are presented in Table 4.

### 7.3. Cheese

The main quality losses of cheese products take place in a storage period and can be summarized as microbial contamination, moisture loss, and the development of off-flavor and other undesirable organoleptic properties [216]. It is very important to underline that the addition of an extra calcium with the aim of crosslinking is not an essential step in cheese coating due to possessing calcium itself [138].

Edible coatings and films have been applied and studied in several types of cheese as packaging system to prevent the quality losses. Zhong, et al. [217] investigated the performance of three different coating materials (i.e., sodium alginate, chitosan, soy protein isolate) with four various application methods on mozzarella cheese. The results showed that alginate-coated samples possessed better overall qualities due to its better wettability on the product surface [217]. Lucera, Mastromatteo, Conte, Zambrini, Faccia, and Del Nobile [137] demonstrated that the antimicrobial activity of potassium sorbate, sodium benzoate, calcium lactate, and calcium ascorbate, which were incorporated into an alginate coating and used to coat mozzarella cheese, were similar. The main difference arose from the sensory point of view. Kavas, Kavas, and Saygili [136] fortified the composite coating of alginate-whey protein isolate coatings with ginger EO and obtained kashar cheese with a lower acidity and higher fat level.

### 7.4. Only Coating/Film, Without Food Application

Alginate is an acidic anionic polysaccharide and able to form covalent bonds and charge-charge electrostatic complexes with protein [218,219]. Especially following heat denaturation of the protein, stronger interactions are formed [218,219]. Studies on alginate-protein interactions and other investigations on alginate-based edible coatings and films (without their application on food products) are presented in Table 5.

## 8. Transport Mechanisms

Ideal edible coatings and films should create a barrier to impede the loss of water vapor, flavor volatiles, and the exchange of CO_2_ and O_2_, in other words, to control the rate of transport of the food products’ molecular components [96]. In this way, the adverse reactions with deteriorative effects (such as respiration and ethylene production) can be diminished or slowed down [96,227,228]. Mass transfer and barrier characteristics are one of the most important properties of edible films and coatings due to their enormous impact on product quality [229]. Mass transport properties of polymer films can be represented by three coefficients: the diffusion coefficient, solubility coefficient, and permeability coefficient, which have been described in detail by Miller and Krochta [230], Donhowe and Fennema [12], McHugh and Krochta [231].

### 8.1. Moisture Barrier Applications

Moisture barrier characteristics (i.e., moisture content, water vapor permeability (WVP), water vapor resistance (WVR), and water vapor transmission rate (WVTR)) have been commonly studied in the literature of alginate-based edible films and coatings since moisture barrier properties are very critical in designing the coating process. In raw, untreated fruits and vegetables, epidermal cell layer and cuticles reduce weight loss and edible coatings and films created an additional extra barrier layer on the stomata and decrease transpiration (and, therefore, weight loss) [90].

The primary mechanism of moisture loss from food product is the vapor-phase diffusion, which was impelled by the water vapor pressure difference between the product and the surrounding air [90,197]. The thickness of the formed film, moisture permeability, temperature, and relative humidity of the surrounding medium are important factors in defining the mass transfer rate [232].

As a simple method, juice leakages from fresh-cut pineapples [158], garlic bulbs [199], ber fruits [165], blueberries [194], fresh-cut watermelon [110], apples [107], plums [91], kilka fish [214], kashar cheese [136], low-fat cut cheese [138], ground beef patties [149] were significantly reduced with the alginate coating application in moisture loss (or weight loss) experiments conducted very often in studies of alginate coated food products.

WVP is another topic that has been widely studied. For the edible films and coatings formed from hydrophilic components, increasing the water activity (a_w_) causes an increase in film moisture content and WVP, due to the swelling of the network with water [8].

Incorporation of additives can alter the moisture transfer properties of the formed film. Various studies showed that lipid addition into the coating formulation helped to decrease the moisture transport [8,39,147,159,191]. The amount of decrease depends on the type, amount, and chain size of the lipid added in the formulation [183]. Pranoto, Salokhe, and Rakshit [142] observed that the addition of 0.4% garlic oil into alginate film formulation decreased WVP significantly. WVR of sunflower oil incorporated alginate coating on fresh-cut apples [82] fresh-cut melon [191], fresh cut pears [147] were determined. Tapia, Rojas-Graü, Rodríguez, Ramírez, Carmona, and Martin-Belloso [161] compared the WVP of both alginate films and coatings on fresh-cut apples and papayas and presented the effect of the sunflower oil addition to the formulations. On the other hand, the addition of plasticizer to the formulation may increase the WVP values of the film due to reducing the intermolecular bonds between polymer chains [39,80,159]. {Olivas, 2008 #108} and Jost, Kobsik, Schmid, and Noller [80] determined the effect of different plasticizers (glycerol, sorbitol [58,80], PEG-8000 (polyethylene glycol), fructose [58]) on WVP.

There has been an increasing interest in using EOs as antimicrobial agents in alginate coating/film formulations. Rojas-Graü, et al. [233] reported that the addition of plant essential oils did not modify the WVP of alginate-apple puree film. Similar results were presented by Norajit, Kim, and Ryu [154] for ginseng extract incorporated alginate films that no significant effect on WVP was observed. On the contrary, Artiga-Artigas, Acevedo-Fani and Martín-Belloso [138] determined that oregano EO incorporated alginate-mandarin fiber coated low-fat cut cheese exhibited higher WVR values than the uncoated samples and the effect increased with increasing concentrations of EO. Kavas, Kavas, and Saygili [136] fortified alginate-whey protein isolate coating with a 1.5% ginger EO and coated kashar cheese. 

The water vapor barrier properties of alginate films were intended to be improved with the addition of other gel-forming biopolymers. Parris, Coffin, Joubran, and Pessen [81] developed alginate-milk based materials (i.e., whole or non-fat milk, whey, sodium caseinate). Films containing whole milk had decreased WVP up to 35% [81]. Likewise, Coughlan, Shaw, Kerry, and Kerry [221] have demonstrated that films formed from alginate-whey protein complexes had lower WVP. Rhim, Wu, Weller, and Schnepf [220] found that up to 10% PGA addition decreased the WVP of soy protein isolate films. Han and Wang [143] evaluated the WVP of sodium alginate-CMC films containing pyrogallic acid.

The type of film formation method also affects water barrier properties. Poverenov, Danino, Horev, Granit, Vinokur, and Rodov [174] demonstrated that LbL-coated melons (with the alginate-chitosan combination) had superior water vapor barrier properties compared to uncoated, only-alginate or only-chitosan coated samples.

Mathematical models were also created to predict the water transport properties of alginate-based edible coatings and films [234].

### 8.2. Gaseous Barrier Applications

Coatings act as barriers to gas exchange, reduce O_2_ uptake and CO_2_ production (in other words, respiration) by the fruit and create a modified atmosphere [90,197]. Still, the coating designer should keep in mind that the modified atmosphere should not create anaerobic conditions to induce the anaerobic growth in the product [96].

Gas barrier properties (especially O_2_) are the second frequently studied transport mechanism. Capillary diffusion (predominant in porous, imperfect materials) and activated diffusion (includes solubilization of the gas in the film, diffusion through the film and release at the other side of the film) are the two mechanisms that occur in gas transport [12]. Transport rates of O_2_ and CO_2_ are strongly influenced by RH at which product is stored [10,96,235]. An increase in RH causes more interaction between water and film/coating molecules, which leads to a plasticized structure that favored mass transfer [235]. Therefore, the permeability characteristics of a coating on a fresh-cut surface are very difficult to predict due to the high RH of the surface [96]. For edible films made from hydrophilic gelling matrices, a higher a_w_ promotes both gas diffusivity and gas solubility due to the water solubility of these gases and, therefore, the gas permeability properties increase [8].

The other influential factor is temperature; high storage temperatures cause the respiration rate increase [96]. For instance, in case the storage temperature increases during the later stages of the product storage, the created MA by the coating/packaging can cause anaerobic respiration [96].

Polarity and the structure of the film also affect the gas transfer properties. More polar films have a more ordered (less porous) structure, therefore, the film becomes less permeable to oxygen, with a high affinity to moisture [76].

The affinity of the fat compounds for oxygen leads to an increase in permeability [159]. Nevertheless, Azarakhsh et al. determined that alginate-sunflower oil coated fresh-cut pineapple pieces had a lower respiration rate due to an increasing internal CO_2_ and decreasing O_2_ concentration [83] and incorporation of lemongrass to the formulation, increased this effect even more [111]. Rojas-Graü, Avena-Bustillos, Olsen, Friedman, Henika, Martín-Belloso, Pan and McHugh [233] noted that the oxygen barrier properties of alginate-apple puree films were not affected by the addition of plant essential oils.

Jost, Kobsik, Schmid, and Noller [80] found that the oxygen permeability of alginate films increased with increasing concentration of glycerol, while the incorporation of sorbitol did not significantly change the gas barrier properties.

Earle and McKee [236] patented an alginate-based coating with O_2_ barrier properties, particularly for dough products with fillings.

Han and Wang [143] analyzed the O_2_ permeability of a sodium alginate-CMC film containing pyrogallic acid.

Buonocore, Conte, and Del Nobile [234] presented a mathematical model, which was fitted to the experimental data of oxygen barrier properties of the alginate-based film.

The respiration rate of alginate coated sweet cherry fruit [90], strawberry [192], peach [197], apples [107,166], pineapples [83], guava [146], and lettuce [200] were evaluated in the literature. Due to being a hydrocolloid, the alginate-based films and coatings generally decreased the respiration rate during storage and, in this way, achieve retention of the quality attributes of the food products. Sipahi, Castell-Perez, Moreira, Gomes, and Castillo [110] stated that the LbL coating application method inhibited the respiration process of fresh-cut watermelon.

Another important change is the amount and/or presence of some internal volatiles of anaerobic conditions, which is created by the high gas barrier properties of the edible coating or film [188]. For instance, higher acetaldehyde and ethanol production compared to the uncoated samples indicate the presence of a modified atmosphere in the alginate coated fruits [79].

### 8.3. Active Compound Release Applications

The edible coating containing active compounds could be very efficient by maintaining a higher concentration of the target compound with a slow migration for an extended period of storage time. Although diffusion of small molecules (such as ethanol and glycerol) is only influenced by the pore size of the matrix, diffusion of larger molecules (such as proteins) from the gel matrix is influenced by the molecular weight of the substance [40,237,238].

The control of the release process with well-determined release rates and migration amounts are very crucial, especially in biotechnology. For this reason, various studies were conducted in the literature. Diffusion of low molecular weight substances such as glucose, L-tryptophan, and α-lactoalbumin and higher molecular weight substances such as albumin, γ-globulun, and fibrinogen [237], and insulin [238] into and from the gel beads were characterized. The release rates of hemoglobin [57,61] and nicotinamide adenine dinucleotide (NAD) [57], as well as the permeability of immunoglobulin G [239] from various alginate films/gels/coatings, were examined. Alginate concentration, α-l-guluronic acid content, the charge of the protein, and the isoelectric point of the protein affected the diffusion rates [51].

Wang and Zhang Newby [240] showed that LbL polyelectrolyte alginate microgels significantly retard the release of small hydrophilic molecules (MW < 250 g/mol).

Zactiti and Kieckbusch modeled permeability [140] and the release of potassium sorbate [71] from alginate films and evaluated the effect of three levels of alginate crosslinking (with different calcium chloride concentrations) on the permeability and release model. As the calcium ion concentration increased, which increased the degree of crosslinking, the mobility of the active substance was prevented and the permeability constant decreased. Moreover, an increase in the sorbate concentration also caused an increase in the permeability values.

Wong, et al. [241] measured the permeability properties of calcium alginate films, which were prepared with the in situ gelation method or the cooling of hot gels when small molecule preservatives sorbate and ascorbate incorporated into the film formulation.

Bustos, Alberti, and Matiacevich [139] evaluated the release parameters of microencapsulated lemongrass oil from the alginate matrix. For this purpose, researchers began with the microencapsulation process, followed by the film preparation and analysis of the release kinetics from the films with the help of assessing antimicrobial activity against *E. coli*.

Transport parameters of n-hexanal [159,160] and D-limonene [160] were determined. Aroma compounds preferably interacted with fat compounds [159]. Therefore, the permeability of aroma compounds is dependent on the interactions between the aroma compounds and film matrices [160].

## 9. Future Trends

Studies about film forming and coating food products with edible biopolymers have been expanded recently. Edible/biodegradable films and coatings can be used to maintain the quality during the shelf life of the product. Promising results have been achieved on fresh cut fruits, vegetables, and meat products coated with alginate solutions with incorporated additives. However, further improvements could be obtained by incorporating new antimicrobial, antioxidative, antibrowning agents to enhance food safety and food quality. A better understanding of any synergistic effect among alginate coating and active agents can be developed.

Blending of film-forming biopolymers to improve the properties of the structure is also a promising strategy. Developing new synergistic gelling systems can be identified as another research gap.

Diffusion properties of active substances from the alginate gel matrix and its structure can be studied in more detailed with varying the concentrations of alginate and cross-linking agents. Comparative studies can be conducted.

The effects of different calcium salts (e.g., calcium chloride, calcium lactate, calcium gluconate) on the quality parameters can be identified in detail with clarified mechanisms.

Most studies on alginate coating applications have been conducted at the laboratory scale and commercial applications are still very limited. Further research with practical applications should focus on the industrial implementation to commercialize the alginate coated food products with increased shelf life. Coating application methods can be readjusted so as to implement a recycle process that does not waste too much of coating solution, decrease microbial load of the solution during recycling, design spraying method for irregular surfaces, design industrial size vacuum tanks, etc. to prevent the disadvantages of the application methods. Therefore, sodium alginate-based edible films and coatings could be used to an even greater extent than they are currently.

## 10. Conclusions

In an ideal case, an edible coating or film should decrease the evaporation of the water content, loss of desirable odor and flavor volatiles, prevent microorganism growth, suppress respiration, and gas exchange; while the modified atmosphere created by the barrier should not cause anaerobic respiration (and, therefore, anaerobic growth) and undesirable volatiles. On the basis of the evaluation of the previous literature on alginate-based edible films and coatings, it can be concluded that the alginate-based edible films and coatings can be efficiently used to accomplish these aims with an enhancing shelf life of fresh-cut fruits and vegetables, meat, poultry, seafood, and cheese. The information summarized here can lead researchers to design successful coating applications.

## Figures and Tables

**Table 1:** The incorporation of antimicrobials in alginate-based edible films and coatings.

Food	Coating/Crosslinking	Antimicrobial	Result	Source
fresh-cut apple	alginate-apple puree/CaCl_2_ (EC ^1^)	oregano, lemongrass, vanillin	high concentrations of Eos ^1^ inhibited the growth of *Listeria innocua*, psychrophilic aerobic bacteria, yeasts, and molds.	Rojas-Graü, et al. [106]
fresh-cut apple	alginate/CaCl_2_ (EC)	thyme oil	15 EOs were evaluated. EC-thyme oil significantly inhibited the TPC ^1^, total coliform, LAB ^1^, yeast and mold growth.	Sarengaowa, et al. [107]
fresh-cut melon [108], apple [109]	alginate/calcium lactate (EC)	malic acid, cinnamon, palmarosa, lemongrass, clove EOs, and their active compounds	malic acid went through antimicrobial action alone. However, when EOs or their active compounds were incorporated, the effect was increased even further.	Raybaudi-Massilia et al. [108,109]
fresh-cut watermelon	alginate/calcium lactate (EC)	*trans*-cinnamaldehyde	EC-antimicrobial agent was significantly effective against psychrotrophs, coliforms, yeasts, and molds.	Sipahi, et al. [110]
fresh-cut pineapple	alginate, sunflower oil/CaCl_2_ (EC)	lemongrass EO	yeast, mold, and the total plate count were significantly reduced, and the shelf-life was prolonged.	Azarakhsh, et al. [111]
strawberry	alginate (EC)	carvacrol, methyl cinnamate	carvacrol was effective against both *E. coli* and *B. cinereal*, on the other hand, methyl cinnamate inhibited only *B. cinerea*.	Peretto, et al. [112]
strawberry	alginate/CaCl_2_ (EF ^1^)	*Cryptococcus laurentii*	microbial decay due to psychrotrophs, yeasts, and molds was significantly reduced.	Fan, Xu, Wang, Zhang, Sun, Sun, and Zhang [94]
capsicum	alginate/CaCl_2_ (EC)	pomegranate peel extract	EC-pomegranate peel extract possessed antimicrobial and antifungal activities.	Nair, et al. [113]
beef pieces and steak	alginate-maltodextrin/CaCl_2_-CMC (EC)	hypochlorous acid (HOCl)	EC-HOCl had no inhibitory effect, although HOCl inhibited the bacterial growth when treated alone.	Williams, et al. [114]
ground beef	alginate/CaCl_2_ (EC)	nisin, acetic acid, lactic acid, potassium sorbate chelating agents: EDTA ^1^, HMP ^1^	only acetic and lactic acid inhibited *E. coli*. Immobilization in EC enhanced the activity of only some of the antimicrobial agent/combination.	Fang and Tsai [115]
ground beef	alginate/CaCl_2_ (EF)	nisin	load of *Brohothrix thermosphacta* significantly decreased until day 7.	Cutter and Siragusa [116]
beef tissue	alginate/CaCl_2_ (EC)	acetic acid, lactic acid	EC-immobilized acids were more effective in reducing *L. monocytogenes* compared to their direct application. Lactic acid had a higher inhibitory effect against Gram (−) at the same pH.	Siragusa and Dickson [117,118]
chicken fillet	alginate alone or alginate-galbanum gum/CaCl_2_ (EC)	EO of *Ziziphora persica*	alginate coating alone had no microbial inhibition effect. Composite coating and addition of EO to formulation had a significant microbial reduction.	Hamedi, et al. [119]
chicken breast fillet	alginate-maltodextrin/CaCl_2_-CMC ^1^ (EC)	lactoperoxidase enzyme	EC-lactoperoxidase decreased the microbial load of *Enterobacteriaceae*, *P. aeruginosa* and aerobic mesophilic bacteria but had no effect on the LAB.	Yousefi, et al. [120]
chicken thigh meat	alginate-whey protein/CaCl_2_ (EC)	lactoperoxidase enzyme	Antimicrobial effect increased with increasing concentration of the lactoperoxidase.	Molayi, et al. [121]
northern snakehead fish	alginate/CaCl_2_ (EC)	nisin, EDTA	EC did not increase the effectiveness of antimicrobials against TVC ^1^ and TPC.	Lu, et al. [122]
smoked salmon	starch-alginate/calcium gluconate (EF)	two strains of LAB, nisin	EF with LAB strains and nisin inhibited *L. monocytogenes* growth.	Concha-Meyer, et al. [123]
smoked salmon	alginate (EF)	sodium lactate, sodium diacetate, commercial formulation consists of both (Opti.Form)	EC-antimicrobials delayed the growth of *L. monocytogenes* during cold storage [124] and greatly prolonged the microbial shelf life during frozen storage [125].	Neetoo, Ye, and Chen [124] and Ye, Neetoo, and Chen [125]
smoked salmon	alginate/CaCO_3_ (EC)	oyster lysozyme, hen egg white lysozyme, nisin	both EC-oyster and EC-hen egg white lysozyme inhibited *L. monocytogenes* and *S. anatum*. Addition of nisin enhanced the antimicrobial activity.	Datta, et al. [126]
abalone	alginate/CaCl_2_ (EC)	bamboo leaf extract, rosemary extract	EC-rosemary extract enhanced bacterial inhibition. PCA ^1^ was used to correlate between the microbial count and biogenic amines.	Hao, Liu, Sun, Xia, Jia, Li, and Pan [93]
rainbow trout fillet	alginate/CaCl_2_ (EC)	resveratrol	coating with antimicrobial agent decreased bacterial, yeast, and mold growth.	Bazargani-Gilani [127]
silver carp fillet	alginate-CMC/CaCl_2_ (EC)	clove EO	EC-clove EO has antimicrobial activity against *L. monocytogenes*, *S. aureus* and *E. coli*, in a decreasing order. Gram (+) bacteria were more sensitive then Gram (−). Concentration increase had a significant effect.	Jalali, et al. [128]
bighead carp fillet	alginate/CaCl_2_ (EC)	horsemint EO	combined effect of EC-horsemint EO significantly decreased the growth rate of TVC and TPC.	Heydari, et al. [129]
winter flounder (fish)	alginate/CaCl_2_ (EC)	glucose oxidase (GOx)	enzyme-alginate blankets exhibited very low surface pH values.	Field, et al. [130]
sea bass	alginate (EC)	tea polyphenols	EC decreased TVC, the reduction was even higher with the incorporation of tea polyphenols into the coating.	Nie, et al. [131]
sea bass [132], red sea bream [133]	alginate/CaCl_2_ (EC)	ε-polylysine [132], 6-gingerol [133]	EC-ε-polylysine and EC-6-gingerol reduced microbial counts, even more effectively than antimicrobial agent or coating, alone.	Cai et al. [132,133]
sea bass [134], Fior di Latte cheese [135]	alginate/CaCl_2_ (EC)	reuterin produced by *Lactobacillus reuteri*	EC system containing biopreservative *L. reuterin* was designed [135]. EC-reuterin was effective in the improvement of microbiological quality [134,135].	Angiolillo et al. [134,135]
kashar cheese	alginate-whey protein isolate (EC)	ginger EO	EC-ginger EO had a bacteriostatic and bactericidal effect on *E. coli* and *S. aureus*, respectively.	Kavas, et al. [136]
mozzarella	alginate/CaCl_2_ (EC)	potassium sorbate, sodium benzoate, calcium lactate, calcium ascorbate	active compounds showed a similar effect in terms of the growth of *Pseudomonas* spp. and *Enterobacteriaceae*. EC–3% potassium sorbate decreased the growth rate.	Lucera, et al. [137]
low-fat cut cheese	alginate-mandarin fiber (EC)	oregano EO	An oregano EO concentration ≥ 2% was effective against *S. aureus*, psychrophilic bacteria, molds, and yeasts.	Artiga-Artigas, et al. [138]
- ^2^	alginate/CaCO_3_ (EF)	microencapsulated lemongrass oil	release kinetics were studied. *L. monocytogenes* and *E. coli* were successfully inhibited.	Bustos, et al. [139]
- ^2^	alginate/CaCl_2_ (EF)	potassium sorbate	the permeability and release of potassium sorbate were modeled.	Zactiti and Kieckbusch [71,140]
- ^2^	alginate clay bionanocomposite (EF)	marjoram, clove, cinnamon essential oils	nanocomposite EF-Marjoram was the most effective in controlling foodborne pathogens due to possessing a high content of phenolic compounds.	Alboofetileh, et al. [141]
- ^2^	alginate/CaCl_2_ (EF)	garlic oil	the inhibitory effect was dependent on the Gram character and increased in the following order: *S. typhimurium* < *E. coli* < *S. aureus* < *B. cereus*.	Pranoto, et al. [142]
- ^2^	alginate (EF)	lysozyme, nisin, grapefruit seed extract, EDTA	EF with grapefruit seed extract alone or in combination with EDTA showed good antimicrobial protection.	Su Cha, Choi, Chinnan, and Park [95]
- ^2^	alginate-CMC/CaCl_2_ (EF)	pyrogallic acid	EF-pyrogallic acid had significant inhibitory effect against *E. coli* and *S. aureus*.	Han and Wang [143]

^1^ EC: edible coating; EF: edible film; EO: essential oil, EDTA: ethylenediaminetetraacetic acid; HMP: sodium hexametaphosphate; LAB: lactic acid bacteria; TVC: total viable count; TPC: total psychrophilic count; PCA: principal component analysis; CMC: carboxyl methylcellulose. ^2^ No food product was covered.

**Table 2:** The incorporation of antioxidant agents in alginate-based edible films and coatings.

Food	Coating/Crosslinking	Antioxidant	Result	Source
fresh-cut papaya	alginate, sunflower oil/CaCl_2_ (EC ^1^)	ascorbic acid	total ascorbic acid content was almost doubled throughout the storage due to oxygen barrier properties.	Tapia, Rojas-Graü, Carmona, Rodríguez, Soliva-Fortuny, and Martin-Belloso [39]
guava	alginate/CaCl_2_ (EC)	pomegranate peel extract	EC increased the antioxidant activity; the effect was even promoted with the addition of pomegranate peel extract.	Nair, et al. [146]
fresh-cut pears	alginate, sunflower oil/CaCl_2_ (EC)	N-acetylcysteine, glutathione	EC-antioxidant agents had significant antioxidant activities, although EC alone did not.	Oms-Oliu, et al. [147]
sliced carrots	alginate/CaCl_2_ (EC)	citric acid	coating process, when applied together with a modified atmosphere, enhanced the shelf life extension effect.	Amanatidou, et al. [148]
ground beef patties	alginate, starch, stearic acid (EF ^1^)	tocopherols	regardless of their incorporation method, tocopherols were effective. Additionally, tocopherols improved the moisture barrier properties.	Wu, et al. [149]
buffalo meat patties	alginate/CaCl_2_ (EC)	sodium ascorbate, citric acid	EC with antioxidants retarded lipid oxidation.	Chidanandaiah, et al. [150]
chicken fillet	alginate-galbanum gum/CaCl_2_ (EC)	EO of *Ziziphora persica*	both galbaum gum and Ziziphora EO ^1^ have high antioxidant activities due to the high phenolic and flavonoid content.	Hamedi, Kargozari, Shotorbani, Mogadam, and Fahimdanesh [119]
pork chops	alginate, modified starch/CaCl_2_ (EC)	rosemary oleoresin	lipid oxidation was inhibited.	Handley, et al. [151]
bream	alginate/CaCl_2_ (EC)	vitamin C, tea polyphenols	EC decreased TBA ^1^ significantly due to being resistant to oxygen diffusion. Vitamin C was more effective in decreasing lipid oxidation.	Song, et al. [152]
red sea bream	alginate (EC)	6-gingerol	EC and antioxidant alone led to an equal inhibition effect; on the other hand, their combination had minimum lipid oxidation values in terms of TBA.	Cai, Wang, Cao, Lv, and Li [133]
bighead carp fillet	alginate/CaCl_2_ (EC)	horsemint EO	EC caused lower oxidation values after the 8^th^ day of storage; the addition of horsemint EO increased this effect even further.	Heydari, Bavandi, and Javadian [129]
silver carp fillet	alginate/CaCl_2_ (EC)	clove EO	EC-clove EO significantly decreased the lipid oxidation probably due to the combined effect of EO and oxygen barrier properties of the alginate coating.	Jalali, Ariiai, and Fattahi [128]
rainbow trout fillet	alginate/CaCl_2_ (EC)	resveratrol	EC-resveratrol coating reduced lipid oxidation significantly.	Bazargani-Gilani [127]
rainbow trout fillet	alginate-clay nanoparticles/ CaCl_2_ (EC)	lycopene	although the EC-lycopene combination helped decrease the FFA ^1^, other fat oxidation parameters such as peroxide and TBA values could not be significantly decreased.	Ehsani, et al. [153]
sea bass	alginate (EC)	tea polyphenols	EC, tea polyphenols inhibited lipid oxidation when they were applied alone, however, the inhibition was higher in their combination due to the synergistic effect.	Nie, Wang, Wang, Lei, Hong, Huang, and Zhang [131]
- ^2^	alginate/CaCl_2_ (EF)	white, red, and extruded white ginseng extracts	EC-ginseng extract showed good antioxidant activity, which can be even increased with controlling the extrusion process.	Norajit, et al. [154]

^1^ EC: edible coating; EF: edible film; EO: essential oil; TBA: thiobarbituric acid; FFA: free fatty acid. ^2^ No food product was covered.

**Table 3:** The application of alginate coatings on fresh-cut fruits and vegetables.

Food	Effects	Source
fresh-cut apples	Optimum composition of alginate-based EC ^1^ was determined for achieving high water and firmness retention during storage.	Ghavidel, et al. [189]
fresh-cut apples	Shelf life of coated apples were prolonged three times compared to uncoated samples. EC maintained firmness, although it increased fermentative metabolites’ (i.e., acetaldehyde and ethanol) production due to MA ^1^.	Rojas-Graü, Tapia, and Martín-Belloso [79]
fresh-cut apples	Base solution was developed with alginate and 26% apple puree. Ethylene, CO_2_ production, and O_2_ consumption were reduced. However, solely vanillin incorporated formulations could achieve acceptable test scores in contrast with other EOs ^1^.	Rojas-Graü, Raybaudi-Massilia, Soliva-Fortuny, Avena-Bustillos, McHugh, and Martín-Belloso [106]
fresh-cut apples	The soluble solid content was increased; stable browning index, acidity, and firmness levels were achieved due to coating with prebiotics incorporated EC.	Rößle, Brunton, Gormley, Wouters, and Butler [166]
apples	Alginate and gelatin-based coatings not only preserved the freshness of the fruit, but also improved the appearance and attractiveness of the fruit.	Moldão-Martins, et al. [190]
apple pieces	Apple pieces were coated with double layers of polysaccharide/lipid (alginate/acetylated monoglyceride) EC to decrease respiratory activity.	Wong, Tillin, Hudson, and Pavlath [184]
fresh-cut apples	Thyme oil had the highest antimicrobial activity among the tested 15 EOs. Physical, chemical and microbial qualities of coated (thyme incorporated) samples were assessed.	Sarengaowa, Hu, Jiang, Xiu, and Feng [107]
fresh-cut apples [109], fresh-cut melon [108]	The effect of malic acid, EOs and their active compounds on quality characteristics were assessed. Due to the inhibition of microflora, respiration and anaerobic fermentation were decreased. However, physicochemical characteristics of the products were affected differently with respect to the type of EOs and concentrations.	Raybaudi-Massilia et al. [108,109]
fresh-cut melon	Sodium alginate-sunflower oil maintained the firmness, however, the coating could not present good barrier properties against O_2_, CO_2_, ethylene, and could not reduce the loss of vitamin C and microbial load.	Oms-Oliu, et al. [191]
fresh-cut melon	LbL technique with oppositely charged alginate-chitosan presented a superior performance on firmness, gas exchange, and microbial growth.	Poverenov, Danino, Horev, Granit, Vinokur, and Rodov [174]
fresh-cut watermelon	LbL coating did not affect the pH and °Brix but preserved the textural firmness and decreased weight loss.	Sipahi, Castell-Perez, Moreira, Gomes, and Castillo [110]
ber fruit	The quality was retained with the application of the composite edible coating, consisting of sodium alginate and olive oil, enriched with ascorbic and citric acids.	Ramana Rao, Baraiya, Vyas, and Patel [165]
strawberry	The quality of the products was enhanced by implementing a yeast antagonist to the formulation.	Fan, Xu, Wang, Zhang, Sun, Sun, and Zhang [94]
strawberry	Effects of alginate, chitosan, pullulan-based EC on antioxidant enzyme system and quality characteristics were compared. All the polysaccharide-based coatings decreased quality losses and extended shelf life.	Li, et al. [192]
strawberry	Incorporation of carvacrol and methyl cinnamate changed the physical properties of the alginate coatings such as turbidity, transparency, and viscosity, depending on their concentration.	Peretto, Du, Avena-Bustillos, Berrios, Sambo, and McHugh [112]
strawberry	The effectiveness of alginate and soy-based coatings on the pH and vitamin C content of the samples were compared.	Ahmed, et al. [193]
blueberry	Numerous ECs (including alginate) were compared in terms of their ability to control quality losses.	Duan, Wu, Strik, and Zhao [92]
blueberry	Performances of chitosan and alginate coatings were compared. Although alginate coatings promoted firmness, lightness and total phenolic content; yeast and mold growth in the samples were induced.	Chiabrando and Giacalone [194]
cherry	The storability period of the coated products increased from 8 to 16 days with a delay in the post-harvest ripening and maintaining higher amounts of total phenolics and antioxidant activity.	Díaz-Mula, Serrano, and Valero [90]
fresh-cut pear	EC with antibrowning agents (N-acetylcysteine and glutathione) reduced microbial growth, increased vitamin C, and the total phenolic content without affecting the firmness of product.	Oms-Oliu, Soliva-Fortuny, and Martín-Belloso [147]
pear	Alginate coated samples had a higher tensile strength, elongation, and elasticity; on the other hand, they had a lower water loss, pH increase, metabolic activities with maintained firmness and green color.	Moraes, et al. [195]
plums	Particularly 3% alginate coating significantly inhibited ethylene production, softening, acidity and water losses, and slowed down carotenoid and anthocyanin increase (and therefore delayed color change) throughout the storage period of plums.	Valero, Díaz-Mula, Zapata, Guillén, Martínez-Romero, Castillo, and Serrano [91]
fresh-cut papaya	The study consisted of two steps: RSM ^1^ was used to determine the number of ingredients in the formulation in terms of WVR ^1^; the chosen formulations helped to achieve increased firmness. On the contrary of several previous studies, the alginate coating did not affect the respiratory rate and ethylene production.	Tapia, Rojas-Graü, Carmona, Rodríguez, Soliva-Fortuny, and Martin-Belloso [39]
Guava	EC-pomegranate peel extract improved the visual and nutritional parameters with delaying senescence.	Nair, Saxena, and Kaur [146]
mango	EC-ascorbic acid retarded firmness loss improved the phenolics and carotenoids content and sensory scores; however, the antimicrobial efficiency was not significant.	Salinas-Roca, et al. [196]
fresh-cut pineapples	The concentration of ingredients in EC was formulated with the help of RSM [83]. Incorporation of lemongrass EO and ascorbic-citric acid into EC prolonged the shelf life whilst maintaining quality attributes.	Azarakhsh et al. [83], [111]
fresh-cut pineapples	Shelf life of the product was significantly improved.	Montero-Calderón, Rojas-Graü, and Martín-Belloso [158]
Peach	Shelf life was increased with maintaining quality.	Maftoonazad, et al. [197]
tomato	Reduced ethylene production, respiration rate, weight loss, a diminution rate of hue angle values (indicated that ripening was delayed) as well as a higher fruit firmness, TSS (total soluble solids concentration), titratable acidity (TA), organic acids (citric, malic and, ascorbic acids), sugars (glucose and fructose), and sensory scores of coated products were achieved.	Zapata, Guillén, Martínez-Romero, Castillo, Valero, and Serrano [89]
potato strips	Possibility of using the alginate coating and ultrasound process as an alternative to blanching of potato strips were investigated. EC was not effective for diminishing the color changes and microbial load.	Amaral, et al. [198]
garlic bulbs	Natural compound isolated from the garlic skin was added into EC. The effects of coating on the mechanical and barrier properties were demonstrated.	Nussinovitch and Hershko [199]
carrot	A 5- to 7-day shelf-life extension of the coated samples was achieved.	Amanatidou, Slump, Gorris, and Smid [148]
lettuce	1-Methylcyclopropene incorporated EC reduced the discoloration, respiration rate, ethylene synthesis (therefore senescence) of samples.	Tay and Perera [200]

^1^ EC: edible coating; MA: modified atmosphere; EO: essential oil; WVR: water vapor resistance; RSM; Response surface methodology.

**Table 4:** The application of alginate coating on meat, poultry, and seafood products.

Food	Effects	Source
ground beef patties	Incorporation of stearic acid into the modified starch-alginate formulation improved the barrier properties against moisture loss and decreased lipid oxidation. Addition of tocopherols increased these effects.	Wu, Weller, Hamouz, Cuppett, and Schnepf [149]
buffalo meat patties	EC ^1^ significantly improved quality attributes such as overall shear force, TBA ^1^, tyrosine value, and microbial counts, etc.	Chidanandaiah, Keshri, and Sanyal [150]
lamb meat	Alginate-maltodextrin coating crosslinked with CaCl_2_-CMC ^1^ led to a decrease in the total volatile nitrogen for refrigerated meat, there was no statistical difference for frozen meat. Although a decrease in the total count of refrigerated meat was only due to calcium ions in the crosslinking solution, EC achieved psychrophilic bacterial inhibition during the frozen storage.	Koushki et al. [207,208]
pork chops	Composite coating with modified starch-alginate with rosemary oleoresin inhibited lipid oxidation and formation of hexanal, pentane, and total volatiles.	Handley, Ma-Edmonds, Hamouz, Cuppett, Mandigo, and Schnepf [151]
pork cuts	Alginate (>1%), helped to decrease the thawing loss; concentration of Ca^2+^ influenced the tenderness of the meat. Optimum coating conditions were defined as 3% alginate, 7% CaCl_2_ with 5–7 min crosslinking time to diminish thawing loss, TBARS ^1^, and an increase in the total protein solubility.	Yu, et al. [209]
cut-up poultry parts	Water evaporated from coating instead of meat. One thick coating application was more convenient than repeating number of coats due to preventing residual calcium salts from being transferred into the alginate dipping solution and the easiness of pealing.	Mountney and Winter [21]
chicken breast and chicken thigh meat	Lactoperoxidase addition into the alginate-based coating system led to higher bacterial and sensorial quality values of chicken meat. The effect was even increased with the increasing concentration of lactoperoxidase.	Yousefi, Farshidi and Ehsani [120], Molayi, Ehsani, and Yousefi [121]
films/casing for breakfast pork sausages	Study assessed the ability of food polymers including gelatin-sodium alginate blends for the formation of stable packaging film. The optimum processing conditions were presented during the extrusion process [210]. The effects of different oil additions on quality parameters of the films/casings [211] and their usage in the manufacturing of sausages were determined [212].	Liu et al. [210,211,212]
bream	EC reduced the rate of quality losses of bream in terms of water loss, pH, TVB-N ^1^, and K-value. A 5% vitamin C content incorporated coating maintained the best quality and sensory results.	Song, Liu, Shen, You, and Luo [152]
red sea bream	EC-6-gingerol coated products obtained a 20-day shelf life extension.	Cai, Wang, Cao, Lv, and Li [133]
japanese sea bass	The synergistic effect of EC and ε-polylysine helped products to maintain a fresh color and tissue hardness, reduce lipid oxidation, protein degradation, and nucleotide breakdown.	Cai, Cao, Bai and Li [132]
japanese sea bass	EC-tea polyphenols provided the greatest effect on quality (TVB-N, lipid oxidation, protein decomposition) and sensory results compared to their effects alone.	Nie, Wang, Wang, Lei, Hong, Huang, and Zhang [131]
sea bass	New biopreservation coating with the addition of food supplement *Lactobacillus reuteri* and its substrate glycerol to EC was developed. The production and antimicrobial effects of reuterin material were evaluated after 2 different fermentation periods.	Angiolillo, Conte, and Del Nobile [134]
sea bass	Two protective processes: salting application of liquid smoke suspension containing resveratrol and alginate coating were used to enhance the quality. Although the treatment combination was effective in reducing oxidation, it could not inhibit bacterial growth.	Martínez, et al. [213]
rainbow trout	0.2% resveratrol improved the effect of EC with the highest inhibition of chemical changes and microbial growth.	Bazargani-Gilani [127]
rainbow trout	Effects of EC with or without lycopene were investigated in terms of various quality parameters.	Ehsani, Paktarmani, and Yousefi [153]
silver carp fillet	Fillets were coated with alginate-CMC. With the help of the controlled release of clove oil, the coating lead to an 8-day shelf life extension without affecting the sensorial properties.	Jalali, Ariiai, and Fattahi [128]
bighead carp fillet	Lower microbial deterioration and auto-oxidation of fish fillets throughout storage were achieved.	Heydari, Bavandi, and Javadian [129]
kilka fish	Shelf life extension was achieved with an alginate-whey protein coating.	Seyfzadeh, et al. [214]
northern snakehead fillets	Contrary to the previous findings of EC-nisin [115,116], researchers did not find any significant evidence that calcium alginate containing nisin increased the effectiveness of the antimicrobial agent. Nevertheless, inhibition of lipid oxidation, TMA-N ^1^, TVB-N, promoting water barrier properties and sensory scores were achieved.	Lu, Liu, Ye, Wei, and Liu [122]
minced fish patties	EC was applied in a different manner: All the ingredients such as minced fish patties, soy protein concentrate, onions, celery, as well as sodium alginate, were blended. The patties were pre-coated initially with soybean oil and afterward dipped in the CaCl_2_ solution for film formation, which prevented the patties from sticking to surfaces during processing.	Rockower, et al. [215]
abalone	An EC-3.5% rosemary extract was successful for the preservation of the product due to reducing TVB-N, controlling biogenic amines, and maintaining better sensory scores.	Hao, Liu, Sun, Xia, Jia, Li, and Pan [93]

^1^ EC: edible coating; CMC: carboxymethyl cellulose; TBA: thiobarbituric acid; TBARS: thiobarbituric acid reactive substances; TVB-N: total volatile basic nitrogen; TMA-N: trimethylamine nitrogen.

**Table 5:** The studies on alginate-based coatings/films without food application.

Study	Source
The effects of soy isolate-sodium alginate and soy isolate-PGA ^1^ interactions on the functional properties of the film formation were investigated and it was found that protein-polysaccharide interactions enhanced the film-forming properties.	Shih [219]
≈10% PGA addition to the soy protein increased the whiteness and tensile strength and decreased the yellowness, percentage elongation at break, WVP ^1^, and water solubility of the multicomponent EF ^1^.	Rhim, et al. [220]
Films formed from alginate-whey protein complexes had higher tensile strength, elastic modulus, and elongation than whey protein alone.	Coughlan, et al. [221]
Different protein-polysaccharide films were compared in terms of oxygen and WVP, tensile strength, transparency, etc.	Yoo and Krochta [48]
By using a Box-Behnken experimental design and RSM ^1^ (for 3 factors; sodium alginate, low acyl gellan, glycerol concentration), a biofilm formulation was designed in terms of mechanical properties.	González-Cuello, et al. [222]
EF formulation was prepared by mixing sodium alginate with a variable quantity of cashew tree gum. However, tensile strength and water barrier properties were weakened due to the competition between two gel-forming polysaccharides for the interaction with calcium ions in the crosslinking step.	Azeredo, et al. [223]
Gelation of calcium alginate with rice starch and/or rice flour was examined. Coarser and more rigid gel structures with an increased diffusion coefficient, a heterogeneous structural resistance constant, and a decreased gelation rate constant were obtained.	Chrastil [64]
Incorporation of garlic oil as a natural antibacterial agent caused darker, yellowish color formation, reduced tensile strength and elongation at break while garlic oil interfered with the calcium ion interactions due to being incorporated before crosslinking.	Pranoto, Salokhe, and Rakshit [142]
Ginseng extract (white, red and extruded white extract) was inserted into the film formulation and a slight decrease in moisture content, and increase in water solubility, transparency, and alteration in the mechanical properties of EF was observed.	Norajit, Kim and Ryu [154]
A new multilayer film, which contained chitosan in the top layer, ornidazole-incorporated polyvinyl alcohol middle and sodium alginate sublayer, with an enhanced swelling rate, water absorption capacity, control of water vapor transmission, and light transmittance, was developed.	Pei, et al. [224]
Incorporation of pyrogallic acid into the sodium alginate-CMC ^1^ matrix increased the gas, vapor, and UV barrier properties of EF.	Han and Wang [143]
Interactions between encapsulated n-hexanal (aroma compound) and alginate matrix affected the barrier, permeability, and surface properties of emulsified alginate EF.	Hambleton, Debeaufort, Bonnotte, and Voilley [159]
Enzyme activity of pig liver esterase with enhanced encapsulation efficiency (i.e., chitosan coating of alginate beads) was studied.	Pauly, et al. [225]
With a novel approach, the alginate film was designed based on the RGB image analysis and color changes. Alginate surface concentration and surface color were modeled to predict the physical properties of the film with a non-destructive method.	Acevedo, et al. [226]

^1^ EF: edible film; PGA: propyleneglycol alginate, produced with the reaction of alginic acid and propylene oxide; WVP: water vapor permeability; CMC: carboxymethyl cellulose; RSM: response surface methodology.
